# Biome-specific effects of nitrogen and phosphorus on the photosynthetic characteristics of trees at a forest-savanna boundary in Cameroon

**DOI:** 10.1007/s00442-015-3250-5

**Published:** 2015-03-10

**Authors:** Tomas Ferreira Domingues, F. Yoko Ishida, Ted R. Feldpausch, John Grace, Patrick Meir, Gustavo Saiz, Olivier Sene, Franziska Schrodt, Bonaventure Sonké, Herman Taedoumg, Elmar M. Veenendaal, Simon Lewis, Jon Lloyd

**Affiliations:** 1 School of GeoSciences, University of Edinburgh, Edinburgh, UK; 2 Faculdade de Filosofia Ciências e Letras de Ribeirão Preto, Universidade de São Paulo, São Paulo, Brazil; 3 Instituto Nacional de Pesquisas da Amazonia, Manaus, Brazil; 4 Centre for Tropical Environmental and Sustainability Sciences (TESS) and School of Marine and Tropical Biology, James Cook University, Cairns, QLD Australia; 5 School of Geography, University of Leeds, Leeds, UK; 6 College of Life and Environmental Sciences, University of Exeter, Exeter, UK; 7 Centre for Tropical Environmental and Sustainability Sciences (TESS) and School of Earth and Environmental Science, James Cook University, Cairns, QLD Australia; 8 Karlsruhe Institute of Technology, Institute of Meteorology and Climate Research, Garmisch-Partenkirchen, Germany; 9 Department of Biology, University of Yaoundé, Yaoundé, Cameroon; 10 Max Planck Institute for Biogeochemistry, Jena, Germany; 11 iDiv, German Centre for Integrative Biodiversity Research Halle, Jena-Leipzig, Germany; 12 Centre for Ecosystem Studies, University of Wageningen, Wageningen, The Netherlands; 13 Department of Geography, University College London, London, UK; 14 Department of Life Sciences, Imperial College London, Silwood Park Campus, Buckhurst Road, Ascot, Berkshire SL5 7PY UK

**Keywords:** Tropical rain forest, Nutrient, Global change, Terrestrial productivity, Photosynthesis

## Abstract

Photosynthesis/nutrient relationships of proximally growing forest and savanna trees were determined in an ecotonal region of Cameroon (Africa). Although area-based foliar N concentrations were typically lower for savanna trees, there was no difference in photosynthetic rates between the two vegetation formation types. Opposite to N, area-based P concentrations were—on average—slightly lower for forest trees; a dependency of photosynthetic characteristics on foliar P was only evident for savanna trees. Thus savanna trees use N more efficiently than their forest counterparts, but only in the presence of relatively high foliar P. Along with some other recent studies, these results suggest that both N and P are important modulators of woody tropical plant photosynthetic capacities, influencing photosynthetic metabolism in different ways that are also biome specific. Attempts to find simple unifying equations to describe woody tropical vegetation photosynthesis-nutrient relationships are likely to meet with failure, with ecophysiological distinctions between forest and savanna requiring acknowledgement.

**Electronic supplementary material:**

The online version of this article (doi:10.1007/s00442-015-3250-5) contains supplementary material, which is available to authorized users.

## Introduction

Forests and savannas are the dominant vegetation types of tropical regions (Walter and Mueller-Dombois 1971) and differ fundamentally in their structural characteristics and species composition (Torello-Raventos et al. 2013). Tree species associated with forest vs. savanna differ in numerous physiological characteristics, such as fire survivorship (Hoffmann et al. 2009; Ratnam et al. 2011), as well as in their wood and foliar characteristics (Gotsch et al. 2010; Hoffmann et al. 2005; Rossatto et al. 2013; Schrodt et al. 2014). We have, however, an incomplete knowledge on how the species differ in photosynthesis characteristics, especially in relation to nutrient availability. Although it does now seem clear that although tropical forests are more productive and comprise larger C stocks than tropical savannas, they tend to have lower maximum photosynthetic C assimilation rates per area of leaf area (Bloomfield et al. 2014; Domingues et al. 2010; Hoffmann et al. 2005; Rossatto et al. 2013).

Although it is well established that photosynthetic capacity can be modulated by leaf N concentrations (Evans 1989; Field and Mooney 1986), in the tropics, where soils are often old and weathered, P limitation may be more typical (Reich and Oleksyn 2004; Reich et al. 2009), with links to stand-level productivity (Mercado et al. 2011; Quesada et al. 2012). On the basis of several lines of evidence, it has, however, also been suggested that, in contrast to tropical forests, savannas may be more likely to be limited by N than P (Lloyd et al. 2009).

Our earlier work from West Africa demonstrated that, depending on their relative concentrations in the leaf tissue, both Rubisco activity and electron transport activity of African savanna and forest trees can potentially be limited by either N or P (Domingues et al. 2010). But in that study interpretations of forest/savanna differences were complicated by savanna measurements from a wide range of precipitation regimes across soil types that were extremely diverse.

Here we report on work from a naturally occurring forest/savanna mosaic in Central Cameroon where we investigate photosynthetic and associated foliar trait characteristics of trees and shrubs for forest and savanna species growing in close proximity and thus the same climatic regime. We aimed to test the hypotheses that under similar climatic conditions in a zone of transition:

1. Scaling between foliar N and P, and its relationship with photosynthesis, is different for forest and savanna species.
2. Species growing in savannas show more indications of N limitation than forest species (which would, in turn, be more likely to be limited by P).

## Materials and methods

### Study location

Measurements were made during the end of the 2007 wet season (October/December) at the Mbam–Djerem National Park, central Cameroon (Electronic Supplementary Material Fig. S1). The area encompasses a transitional zone between the Guinea–Congo/Sudan formations (Maisels 2004; White 1983) where savannas co-exist with tall canopy forest and gallery forest in a mosaic characterised by relatively sharp boundaries (Mitchard et al. 2009). Mean annual precipitation is estimated at about 1.6 m year^−1^ (Hijmans et al. 2005).

### Study plots

Measurements were made in seven permanent 1-ha plots chosen to contain three vegetation groupings recognizable on the basis of structure and species composition, as classified by Torello-Raventos et al. (2013), viz.: (1) ‘long-grass savanna woodland’ (three plots; MDJ-02, MDJ-04 and MDJ-08); (2) three plots within the broad ‘forest’ groupings (MDJ-01, MDJ-03 and MDJ-07); and finally, (3) ‘transitional forest’ as represented by a single plot (MDJ-05). This plot was once savanna, but has recently been invaded by forest species as described for our study area (Mitchard et al. 2011). For the interested reader, photographs of this site as well as MDJ-04 (long-grass savanna) and the forested MDJ-01 and MDJ-03 are provided in Fig. 6 of Torello-Raventos et al. (2013).

### Site characterisation

Biodiversity indices and measurements of plot structure were determined as detailed in Torello-Raventos et al. (2013). Soil sampling and associated measurements were made as described in Quesada et al. (2010) and Veenendaal et al. (2014).

### Gas exchange characteristics

Sampling leaves on excised upper canopy branches with the assistance of a tree climber, data were obtained from 196 leaves fully exposed to the sun sampled from 69 individuals representing 42 species of adult perennial C3 trees and shrubs. Within each plot the quantitatively dominant species were selected, and measurements made for photosynthetic capacity using a LI-COR-6400 portable photosynthesis system (*A*–*C*_i_ curves at high photon irradiance), leaf nutrients and leaf mass per unit area (*M*_a_). Methodological details follow Domingues et al. (2010), with a simple modification introduced for estimation of the two key photosynthetic capacity parameters ($V_{{{\text{cmax}} }}$, the maximum rate of carboxylation and *J*_max_, the maximum rate of electron transport) optionally incorporating a mesophyll conductance term (*g*_m_) into the parameter estimation routine. The parameter *g*_m_ is difficult to estimate from CO_2_ response curves and the approach adopted in the present work followed two steps. A curve fit based on CO_2_ concentrations at the intercellular air spaces (*C*_i_) as reported in Domingues et al. (2010) was performed first to generate initial values of the photosynthetic capacity parameters ($V_{{cmax_ - C_{i} }}$ and $J_{{max - C_{i} }}$). Next, a second curve fit was performed incorporating *g*_m_ in order to calculate CO_2_ concentrations at the sites of carboxylation (*C*_c_) using the $V_{{cmax_ - C_{i} }}$ and $J_{{{max} - C_{i} }}$ values as a starting point for the iteration process. To make our data comparable, the parameter fits for $V_{{{\text{cmax}}}}$ and *J*_max_, as estimated from *A*–*C*_i_ curves at ambient temperatures (typically 28–33 °C), were scaled to a reference temperature (25 °C) as described in Bernacchi et al. (2001).

Usually three replicates (leaves) were sampled from each individual plant sampled in this study, and up to three, but sometimes one or two individuals of the same species, were sampled at a given plot (Electronic Supplementary Material Table S1). When possible, measurements were taken directly from tree branches, but often branches were detached from trees and smaller stems were then immediately re-cut under water.

### Statistical and modelling analysis

For statistical comparisons of leaf traits among plots, species’ averages within each plot were computed after first taking averages from replicated samples of individual plants. Statistical inferences on the relationships of photosynthetic capacity parameters and associated leaf traits (nutrients and/or structure) were based on both simple and multiple linear regressions using values derived from determinations on individual leaves. Data were log_10_ transformed before standardized major axis (SMA) (Warton et al. 2006) analyses but not before the application of an area version of a dual-limitation model of N and P introduced by Domingues et al. (2010) and here employed on an area basis viz.

1 $$V_{\hbox{max} } \; = \;\hbox{min} \left\{ {\begin{array}{*{20}c} {a_{\text{N}} + b_{\text{N}} [N]_{\text{a}} } \\ {a_{\text{P}} + b_{\text{P}} [P]_{\text{a}} } \\ \end{array} } \right\} ,$$

where *V*_max_ is either $V_{{{\text{cmax}}}}$ or *J*_max_, *a*_N_ and *a*_P_ are intercepts and *b*_N_ and *b*_P_ are slopes empirically derived from fitting the model to the data. Model comparisons were based on evaluations of Akaike information criteria (AIC) and Bayesian information criteria (BIC). Bootstrapping analysis (Chernick and LaBudde 2011) was applied in order to derive confidence intervals for parameters which originated from the application of the dual-limitation model (Eq. 1). All statistical analysis was conducted using the statistical environment R (R Development Core Team 2011).

For these *C*_i_-based analyses of the nutrient dependencies of $V_{{{\text{cmax}}}}$ and *J*_max_ we also included data from the West African transect ZOT in Ghana sampled with an identical methodology (Domingues et al. 2010) so as to increase both the sample size and the variation of N and P observed.

## Results

### Assignment of species to the forest or savanna guilds

**Fig. 1:**
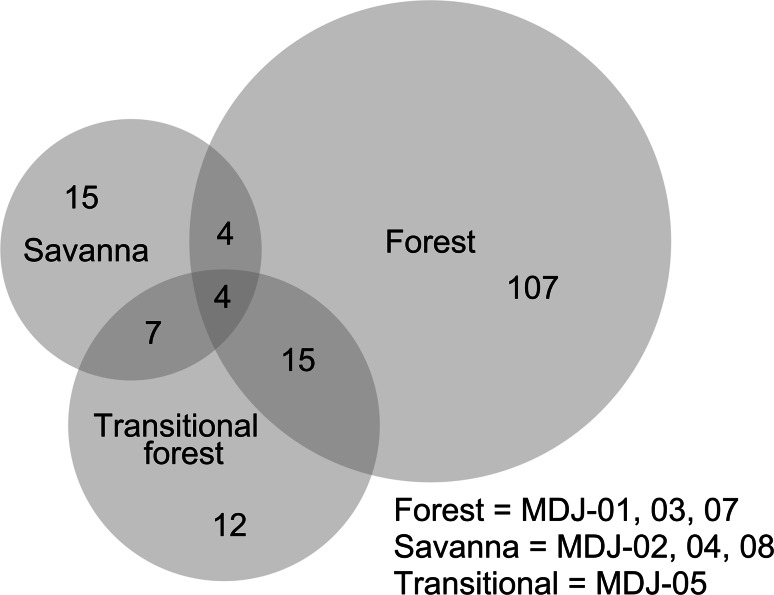
Venn–Euler diagram showing the abundance of tree species across sample plots considered as ‘forest’, ‘savanna’ and ‘transitional forest’ according to the classification of Torello-Raventos et al. (2013). *Numbers* refer to number of individual species

As described in detail by Torello-Raventos et al. (2013), species found within the forest-savanna ecotone can usually be classified as belonging to ‘forest’ or ‘savanna’ based on their observed distribution, although a small degree of overlap inevitably occurs. This is illustrated in Fig. 1 where the distributions of tree/shrub species (stem diameter at breast height > 0.1 m) are represented using a Venn–Euler diagram. Here, the number of plant species found in more than one vegetation type is represented numerically and proportionally by the areas of intersection among the circles. Only eight out of the 164 species observed in the seven study plots occurred in both forest and savanna [see also Table E1 of the Supplementary Information of Torello-Raventos et al. (2013)]. The transitional forest (MDJ-05) did, however, contain many savanna species, as well as several unique species not found in the nearby forest or savanna plots.

### Stand properties

Consistent with Fig. 1, a larger variety of families, genera and species were found at the forest sites (Table 1). Not surprisingly, the forest plots also had larger stem density and larger basal area (Table 1). The transitional forest plot MDJ-05 had the highest stem density but that added to a low total basal area (Table 1). That plot also had a relatively large number of dead standing savanna trees (data not shown).

**Table 1:** Soil and vegetation properties of the study plots

	Forest			Transition	Savanna		
Location	MDJ-01	MDJ-03	MDJ-07	MDJ-05	MDJ-02	MDJ-04	MDJ-08
Latitude	6.1683N	5.984N	6.007N	5.980N	6.164N	5.999N	6.213N
Longitude	12.825E	12.869E	12.887E	12.869E	12.824E	12.868E	12.749E
Biodiversity measures							
Number of Families	25	30	28	21	15	12	12
Number of Genera	43	56	54	32	23	16	19
Number of Species	59	79	69	44	23	19	19
Shannon index	3.05	3.09	3.42	2.53	2.34	2.02	2.15
Vegetation structure							
Basal area, m^2^ ha^−1^	35.6	25.6	25.6	14.0	4.3	5.9	8.1
Canopy area index, m^2^ m^−2^	3.24	2.98	1.75	2.85	0.45	0.36	0.48
Tree density, ha^−1^	611	467	465	684	136	213	241
Soil physical and chemical properties (0.0–0.3 m)							
Sand fraction	0.41	0.65	0.67	0.58	0.28	0.56	0.59
Silt fraction	0.38	0.23	0.16	0.18	0.33	0.16	0.28
Clay fraction	0.22	0.12	0.18	0.25	0.39	0.28	0.13
pH (H_2_O)	6.53	4.88	4.70	4.50	5.32	4.92	5.81
[N], mg g^−1^	1.49	0.71	0.68	0.80	1.56	0.62	0.72
[C], mg g^−1^	19.4	8.6	9.0	12.0	26.5	9.8	11.5
C/N ratio	11.6	12.2	12.6	15.0	17.2	15.7	15.2
Total P, μg g^−1^	977	307	738	576	997	316	364
ECEC, mmol eq kg^−1^	21.1	10.5	7.3	1.6	9.3	5.2	16.4
Leaf traits (mean ± standard deviation)							
* M*_a_, g m^−2^	80 ± 31	97 ± 26	109 ± 21	113 ± 41	136 ± 23	127 ± 26	135 ± 32
* V*_cmax_, μmol m^−2^ s^−1^	39.2 ± 13.6	42.3 ± 12.2	45.7 ± 12.1	44.8 ± 8.3	54.6 ± 11.8	39.2 ± 9.2	27.8 ± 0.1
* J*_max,_ μmol m^−2^ s^−1^	76.8 ± 20.5	79.3 ± 19.5	88.2 ± 17.8	81.0 ± 18.6	87.6 ± 17.5	67.9 ± 12.3	47.5 ± 10.2
* N*_a_, g m^−2^	1.97 ± 0.55	2.26 ± 0.67	2.13 ± 0.46	2.12 ± 0.56	2.67 ± 1.39	1.48 ± 0.27	1.44 ± 0.06
* P*_a_, g m^−2^	0.12 ± 0.05	0.10 ± 0.03	0.09 ± 0.02	0.10 ± 0.03	0.16 ± 0.08	0.13 ± 0.04	0.12 ± 0.07
δ^13^C, ‰	−29.8 ± 1.2	−30.0 ± 1.0	−30.6 ± 1.2	−30.0 ± 0.9	−30.5 ± 0.3	−30.3 ± 0.3	−29.3 ± 0.1

*ECEC* Effective cation exchange capacity, *M*_a_   leaf mass per unit area, *V*_25_  estimated maximum rate of Rubisco limited carboxylation at 25 °C, *J*_25_  estimated maximum rate of electron transport at 25 °C, *N*_a_   leaf nitrogen per unit area, *P*_a_  leaf phosphorus per unit area

Upper layer soil physical and chemical properties (0.0–0.3 m) also varied substantially amongst plots but—with the exception of soil C/N ratio—not consistently between the two main vegetation formation types (Table 1). As an example, effective cation exchange capacity (the sum of exchangeable bases plus Al) was highest at plot MDJ-01 (forest) and MDJ-08 (savanna) with other forest and savanna plots having only about one-third of these values while total soil P varied between 307 and 977 μg g^−1^ for the forest plots and 316 and 997 μg g^−1^ for the savanna plots.

### Leaf traits

**Fig. 2:**
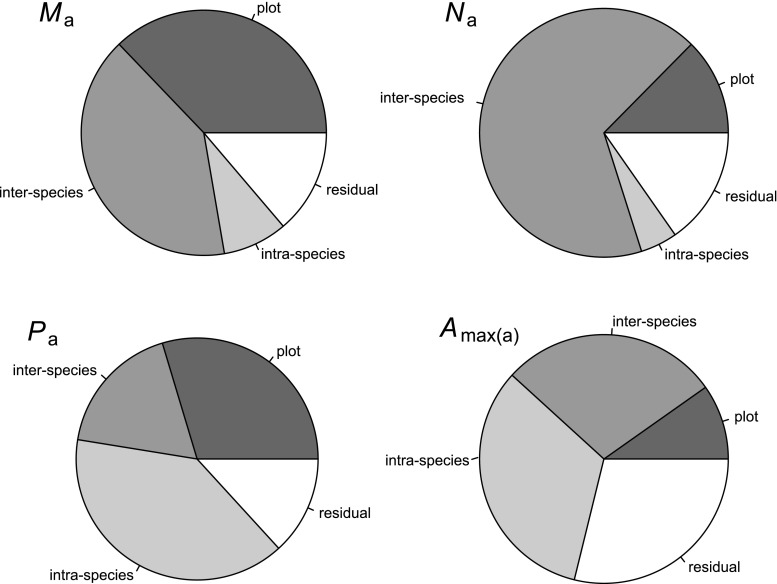
Partitioning of the total variance between plots, species, individual trees. The residual term includes between-leaf variation and experimental error. *M*_a_ Leaf mass per unit area, *N*_a_ N per unit leaf area, *P*_a_ P per unit leaf area, *A*_max(a)_ net CO_2_ assimilation rate per unit leaf area at saturating light and [CO_2_]

A partitioning of the measured trait variation between plots, species, individual trees, and a residual component (representing the average variation between leaves within any given tree plus any experimental error) is shown in Fig. 2. For *M*_a_ and N per unit area (*N*_a_), this shows most of the variation not due to plot location was attributable to species identity, with the proportion of variation between trees of the same species and ‘residual variation’(i.e. attributable to within-tree variability and experimental error) being relatively small. By contrast, for *P*_a_ and the light/CO_2_ saturated assimilation rate (*A*_max(a)_) most of this variation was within species or within individual trees themselves (Fig. 2). In view of this inconsistent pattern of variation among traits, we undertook all analyses on a leaf-wise basis rather than deriving individual tree means or some sort of (often necessarily cross-plot) species’ average value.

**Fig. 3:**
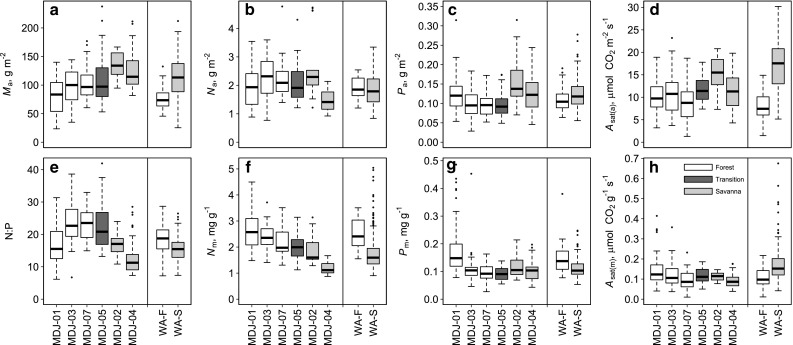
Statistical comparison of leaf attributes of forest (*white bars*), savanna (*light grey*) and transitional forest (*dark grey*) sampled in central Cameroon. Also shown (*right of vertical line*) are forest (*WA-F*) and savanna data (*WA-S*) from a previous study in West Africa (Domingues et al. 2010). **a** *M*_a_; **b** *N*_a_; **c** *P*_a_; **d** net CO_2_ assimilation rate per unit leaf area at saturating light and ambient [CO_2_] [*A*_sat(a)_]; **e** leaf N/P ratio (*N*:*P*; g g^−1^); **f** N per unit leaf mass (*N*_m_); **g** P per unit leaf mass (*P*_m_); **h** net CO_2_ assimilation rate per unit leaf mass area at saturating light and ambient [CO_2_] [*A*_sat(m)_]; for other abbreviations, see Fig. 2

**Fig. 4a–d:**
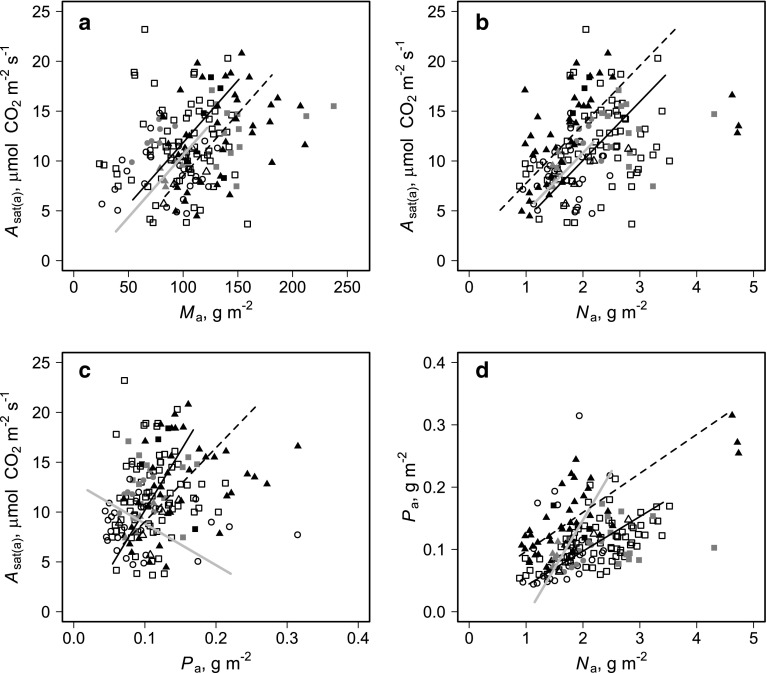
Bivariate plots of observed relationships between area-based measures of light-saturated photosynthetic rate [*A*_sat(a)_], N and P. Deciduous forest (*squares*), evergreen forest (*circles*) and savanna (*triangles*) with* open*, *grey* and *black* symbols indicating individuals in forest, transition and savanna environments, respectively. *Lines* are standard major axis (SMA) regression fits for forest deciduous (*black line*), forest evergreen (*grey line*), and savanna deciduous leaves (*dotted line*)

Despite often considerable overlap between leaf attributes found in forest versus savanna, some differences are striking (Fig. 3). For example, forest leaves typically had a lower *M*_a_, higher *N*_m_, a lower *P*_a_ and a higher N:P ratio. Also shown in Fig. 3 are the equivalent data for forest and savanna from the ZOT component of the West African study of Domingues et al. (2010). This shows some interesting differences, the statistically significant of which are evaluated—along with a comparison for the Cameroon forest species with South American forest—in Table 2. Taken together, Table 2 and Fig. 4 show several intra- and cross-continental differences.

**Table 2:** Comparisons of the main leaf traits (shown as medians) between (1) forest and savanna in Cameroon (this study); (2) and (3) forest and savanna in this study as compared to a previous study in West Africa (Domingues et al. 2010); (4) forest in this study as compared to a previous study in Amazon Basin for which soils has been classified into two fertility groups (Fyllas et al. 2009)

Location	Vegetation formation type	M_a_ (g m^−2^)	N_a_ (g m^−2^)	P_a_ (g m^−2^)	A_sat(a)_ (μmol m^−2^ s^−1^)	N_m_ (mg g^−1^)	P_m_ (mg g^-1^)	*N:P* (g g^−1^)	A_sat(m)_ (μmol ^−1^ s^−1^)
Comparison 1. This study: Forests vs. Savanna									
Cameroon	Forest	95	2.12	0.10	10.3	23.5	1.11	20.9	0.105
Cameroon	Savanna	119^***^	1.63^***^	0.12^**^	11.8 ^ns^	12.8^***^	1.08^*^	13.0^***^	0.085^*^
Comparison 2. Forests: Cameroon vs. West Africa									
Cameroon	Forest	95	2.12	0.10	10.3	23.5	1.11	20.9	0.105
West Africa	Forest	75^***^	1.76^**^	0.11 ^ns^	8.6^***^	24.7 ^ns^	1.37^***^	18.6^*^	0.105 ^ns^
Comparison 3. Savanna: Cameroon vs. West Africa									
Cameroon	Savanna	119	1.63	0.12	11.8	12.8	1.08	13.0	0.085
West Africa	Savanna	92^***^	1.73 ^ns^	0.14 ^ns^	9.8 ^ns^	18.5^***^	1.68^***^	12.7 ^ns^	0.107^*^
Comparison 4. Forest: Cameroon vs. Amazon Basin									
Cameroon	Forest	95	2.12	0.10	10.3	23.5	1.11	20.9	0.105
Amazon “low nutrient soil”	Forest	97	1.90^**^	0.06^***^	ND	20.1^***^	0.70***	28.9^***^	ND
Amazon “high nutrient soil”	Forest	95 ^ns^	2.09 ^ns^	0.11 ^ns^	ND	21.6^**^	1.11 ^ns^	19.4^*^	ND

*M*_a_   leaf mass per unit area, *N*_a_   nitrogen per unit leaf area, *P*_a_   phosphorus per unit leaf area, *A*_sat(a)_  Net CO_2_ assimilation rate per unit leaf area at saturating light and ambient [CO_2_], *A*_sat(m)_   Net CO_2_ assimilation rate per unit leaf mass area at saturating light and ambient [CO_2_], *N*_m_   nitrogen per unit leaf mass, *P*_m_   phosphorus per unit leaf mass, *N*:*P* leaf nitrogen/phosphorus ratio (g g^−1^)

*Asterisks* denote the result of a Kruskal-Wallace test of significance (* *p* < 0.05, ** *p* < 0.01,*** *p* < 0.001)

Focussing first on the current study (comparison 1) forest (${\mathbb{F}}$) leaves had a higher N content than savanna (${\mathbb{S}}$) leaves on both an area and a mass basis. The effect of vegetation formation type (${\mathbb{V}}$) on foliar P contents was, however, rather small. Despite the differences between ${\mathbb{F}}$ and ${\mathbb{S}}$ in both *N*_a_ and *P*_a_ (which we also point out were of opposite sign), there was no effect of ${\mathbb{V}}$ on *A*_sat(a)_ (Table 2: comparison 1).

Also in Table 2 (comparison 2) we compare the significance of differences between forest leaves from this study in Cameroon with the earlier study from West Africa. With the same methodology we find West African ZOT forest leaves to have had significantly lower *M*_a_, *N*_a_ and *A*_sat(a)_ than for Cameroon, but with about the same P, N and *A*_sat_ on a mass basis.

The corresponding comparison for savanna also showed West African leaves to have a lower *M*_a_, but with their N concentration and photosynthetic rates on a mass basis no lower than for Cameroon. Nevertheless, because of the lower *M*_a_ in West Africa, *A*_sat(m)_ were higher; this was also associated with higher *P*_m_ and *N*_m_ (Table 2).

Finally, to help gain a broader perspective we compared our Cameroon forest data with those obtained from the forests of the Amazon Basin (Fyllas et al. 2009, 2012). The latter study classified Amazonian sites into two soil fertility groups on the basis of their soil chemical properties and demonstrated that ‘low nutrient soil’ forests have lower leaf N and P on both an area and mass basis, and also higher N:P ratios than those of our Cameroon study area (Table 2). On the other hand, leaves from sites classified as ‘high nutrient soil’ by Fyllas et al. (2009) were very similar in composition to our Cameroon sites on both a mass and area basis. We therefore conclude that the African forest species sampled here are quite similar in their N and P concentrations to trees occurring on the more fertile soils of the Amazon Basin. As *M*_a_ are, on average, similar, this is true on both an area and mass basis, with the African forests studied here differing from the Amazon Basin forests on lower nutrient status soils mostly in terms of a higher foliar P status.

### Bivariate relationships

Concentrating again on the Cameroon data, Fig. 4 shows the associations between *A*_sat(a)_, *M*_a_, *N*_a_ and *P*_a_ where-noting that all savanna species were deciduous—we have further differentiated forest species according to their leaf habit (evergreen vs. deciduous) as in Schrodt et al. (2014).

Although the relationship between *A*_max(a)_ and *M*_a_ was not significant for either of the forest habit types, for the deciduous savanna species (${\mathbb{S}}_{\text d}$), there was a statistically significant positive relationship (*p* = 0.038). Compared to the two forest types there was a (common) SMA slope of 0.13 μmol CO_2_ g^−1^ dry weight s^−1^ but with a clear difference in intercept (Fig. 4a). That is to say, for any given *M*_a_, ${\mathbb{S}}_{\text d}$ tend to have a consistently lower *A*_sat(a)_ than either of the forest habit types. When examined as a function of *N*_a_ (Fig. 4b), all three habitat groupings showed statistically significant relationships (*p* < 0.10) with ${\mathbb{S}}_{\text d}$ having a photosynthetic rate about 2.5 μmol CO_2_ m^−2 ^s^−1^ greater than the forest species at any given *N*_a_ (common slope of 5.90 μmol CO_2 _g^−1^ N s^−1^). Relationships between *A*_sat(a)_ and *P*_a_were significant only for ${\mathbb{S}}_{\text d}$ (slope = 74 μmol CO_2_ g^−1^ P s^−1^; *p* < 0.05), with data for the forest evergreen leaves (${\mathbb{F}}_{\text e}$) even suggesting a (non-significant) negative relationship (Fig. 4c). The *N*_a_:*P*_a_ relationship was statistically significant for both deciduous types, with a clear difference in elevation: at any given *N*_a_, ${\mathbb{S}}_{\text d}$ typically had a *P*_a_ about 0.04 g m^−2^ greater than their deciduous forest counterparts (${\mathbb{F}}_{\text d}$) (Fig. 4d).

For all four bivariate relationships investigated there were no clear indications of trees sampled from transitional vegetation being distinct from those of either the (non-transitional) forest or savanna vegetation types. Overall, we conclude from Fig. 4 that the strongest relationship is between *A*_sat(a)_ and *N*_a_ with deciduous savanna species were markedly different from both forest types: in particular, they exhibited a substantially higher mean *A*_sat(a)_ for any given *N*_a_. Moreover, unlike forest species, for savanna species there is also a dependence of *A*_sat(a)_ on *P*_a_. At any given *N*_a_, *P*_a_ was higher in the leaves of savanna trees.

### Variations in Rubisco and electron transport capacities in relation to N and P

Although as noted in the Materials and methods one would ideally like to model variations in both $V_{{{\text{cmax}}}}$ and *J*_max_ in terms of the partial pressure of CO_2_ in the chloroplast (*C*_c_), this requires some reliable measure of the leaf ‘internal’ conductance (*g*_m_). Nevertheless, for the study here, both $V_{{{\text{cmax}}}}$ and *J*_max_ were eventually simply estimated from the *A*–*C*_i_ curve with the associated kinetic constants for *g*_m_ = ∞ applied (Von Caemmerer 2000). This decision was made on the basis of: (1) there being no significant relationship between our curve-fitting-derived estimates of *g*_m_ and traits previously considered to covary with it [viz*. M*_a_ or δ^13^C (Niinemets (1999)]; (2) there being no consistent differences in apparent *g*_m_ between vegetation types; and (3) there being little systematic difference observed between *C*_c_- and *C*_i_- (intercellular spaces) based estimates of these photosynthetic parameters (Electronic Supplementary Material Fig. S1).

Details of area-based photosynthesis-nutrient relationships so derived for simple ordinary least squares (OLS) linear models and the more complex dual-limitation model of Eq. 1 are shown in Table 3. For F_e_ the best fit according to the AIC was the simple linear model wherein *V*_25_ is a simple function of *N*_a_ (*r* ^2^ = 0.17, *p* = 0.002), with the BIC—similar to the AIC but with more severe penalties for extra terms—giving the same result. Of the linear models, a simple dependence of *V*_25_ on *N*_a_ also gave the best fit for ${\mathbb{F}}_{\text d}$ according to the BIC (*r* ^2^ = 0.21, *p* = 0.001) but with the alternative dual-limitation model (Domingues et al. 2010) being marginally better according to the AIC (*r* ^2^ = 0.23, *p* = 0.001). Note, however, that in this model the *P*_a_ term is negative, implying an inhibitory effect of P on *V*_25_. Overall, the results for the two forest types were similar, so when they were combined there was, not surprisingly, an increase in the correlation coefficient values for the *N*_a_-based models with the AIC suggesting the dual-limitation model (*r* ^2^ = 0.29) to be marginally superior to the simple *N*_a_-based linear model (which was in turn unambiguously favoured when considering the BIC). Note that in no case was there any indication for a role for *P*_a_ as a modulator of *V*_25_ when considered on its own (*r* ^2 ^≤ 0.01) for forest trees, with *P*_a_ having only a marginal influence when considered in conjunction with *N*_a_.

**Table 3:** Comparisons of predictive models of area based maximum carboxylation capacity (*V*_25_ = *V*_cmax_-area 25 °C; μmol m^−2^ s^−1^) based on leaf nitrogen and/or phosphorus content

Equation	*r* ^2^	AIC	BIC	*p*
Forest evergreen				
* V*_25_ = 16.43 + 11.72*N*_a_	**0.17**	**371.35**	**377.14**	0.002
* V*_25_ = 35.53 + 19.03 *P*_a_	0.01	381.21	387.01	0.495
* V*_25_ = 16.49 + 12.50*N*_a_ – 14.64 *P*_a_	0.15	373.05	380.77	0.007
* V*_25_ = 28.22 + 5.82*N*_a_ – 134.72 *P*_a_ + 65.97*N*_a_ *P*_a_	0.15	374.04	383.70	0.013
* V*_25_ = min(16.43 + 11.72*N*_a_; 50.23 + 24.52 *P*_a_)	0.15	374.35	385.01	0.037
Forest deciduous				
* V*_25_ = 23.62 + 10.41*N*_a_	0.21	830.27	**838.23**	<0.001
* V*_25_ = 42.80 + 39.09 *P*_a_	0.00	854.61	862.58	0.323
* V*_25_ = 25.50 + 10.91*N*_a_ – 26.49 *P*_a_	0.20	831.75	842.36	<0.001
* V*_25_ = 7.71 + 19.37*N*_a_ + 143.95 *P*_a_ – 78.49*N*_a_ *P*_a_	0.21	831.76	845.03	<0.001
* V*_25_ = min(16.77 + 14.00*N*_a_; 57.83 – 32.13 *P*_a_)	**0.23**	**829.26**	843.53	<0.001
Forest (evergreen and deciduous)				
* V*_25_ = 18.61 + 12.03*N*_a_	0.27	1208.24	**1217.39**	<0.001
* V*_25_ = 38.95 + 46.60 *P*_a_	0.01	1255.86	1265.01	0.080
* V*_25_ = 19.60 + 12.45*N*_a_ – 17.26 *P*_a_	0.27	1209.73	1221.93	<0.001
* V*_25_ = 11.02 + 16.96*N*_a_ + 64.75 *P*_a_ – 41.37*N*_a_ *P*_a_	0.27	1210.75	1226.00	<0.001
* V*_25_ = min(12.83 + 15.22*N*_a_; 57.43 – 34.33 *P*_a_)	**0.29**	**1206.40**	1222.65	<0.001
Savanna (deciduous)				
* V*_25_ = 26.22 + 8.25*N*_a_	0.19	779.04	786.89	<0.001
* V*_25_ = 25.30 + 111.17 *P*_a_	0.18	780.01	787.86	<0.001
* V*_25_ = 22.20 + 5.27*N*_a_ + 66.68 *P*_a_	0.23	775.52	785.98	<0.001
* V*_25_ = 1.57 + 17.67*N*_a_ + 167.62 *P*_a_ – 57.46*N*_a_ *P*_a_	0.27	770.21	783.29	<0.001
* V*_25_ = min(34.87 + 5.83*N*_a_; 9.79 + 251.86 *P*_a_)	**0.30**	**765.86**	**779.94**	<0.001
Forest and savanna (deciduous and evergreen)				
* V*_25_ = 22.13 + 10.46*N*_a_	0.26	2151.39	2162.29	<0.001
* V*_25_ = 36.03 + 57.33 *P*_a_	0.04	2223.43	2234.33	0.001
* V*_25_ = 21.53 + 10.24*N*_a_ + 8.83 *P*_a_	0.25	2153.07	2167.60	<0.001
* V*_25_ = 6.44 + 17.70*N*_a_ + 118.70 *P*_a_ – 51.98*N*_a_ *P*_a_	**0.29**	**2141.92**	**2160.10**	<0.001
* V*_25_ = min(22.13 + 10.46*N*_a_; 75.85 + 19.52 *P*_a_)	0.25	2154.39	2173.56	<0.001

*N*_a_   nitrogen per unit leaf area (g m^–2^), *P*_a_   phosphorus per unit leaf area (g m^−2^)

Models addressed in detail in the “Results” and “Discussion” sections are highlighted in bold

**Table 4:** Comparisons of predictive models of area based maximum electron transport rate (*J*_25_  = *J*_max_-area 25 °C; μmol m^−2^ s^−1^) based on leaf nitrogen and/or phosphorus content

		*r* ^2^	AIC		BIC	*p*
Forest evergreen						
* J*_25_ = 32.69 + 21.14*N*_a_		**0.18**	**426.88**		**432.68**	0.001
* J*_25_ = 64.15 + 64.28[*P*]_a_		0.02	436.24		442.03	0.181
* J*_25_ = 32.66 + 20.68*N*_a_ + 8.58*P*_a_		0.16	428.85		436.58	0.005
* J*_25_ = 28.78 + 22.89*N*_a_ + 48.23*P*_a_ – 21.78*N*_a_ *P*_a_		0.15	430.81		440.47	0.015
* J*_25_ = min(32.69 + 21.14*N*_a_; 78.99 + 64.40*P*_a_)		0.16	429.88		440.54	0.028
Forest deciduous						
* J*_25_ = 40.42 + 19.60*N*_a_		0.24	946.15		**954.12**	<0.001
* J*_25_ = 83.76 – 9.89*P*_a_		−0.01	975.38		983.34	0.888
* J*_25_ = 49.03 + 21.89*N*_a_ – 121.73*P*_a_		0.25	944.48		955.10	<0.001
* J*_25_ = 25.94 + 32.89*N*_a_ + 99.60*P*_a_ – 101.93*N*_a_ *P*_a_		0.26	945.34		958.61	<0.001
* J*_25_ = min(30.05 + 24.93*N*_a_; 105.64 – 55.54*P*_a_)		**0.26**	**945.12**		959.39	<0.001
Forest (evergreen and deciduous)						
* J*_25_ = 35.21 + 21.30*N*_a_		0.29	1376.58		**1385.73**	<0.001
* J*_25_ = 73.34 + 62.94*P*_a_		0.01	1428.19		1437.34	0.172
* J*_25_ = 38.25 + 22.58*N*_a_ – 52.86*P*_a_		0.29	1376.94		1389.14	<0.001
* J*_25_ = 15.48 + 34.54*N*_a_ + 164.81*P*_a_ – 109.80*N*_a_ *P*_a_		0.29	1376.58		1391.83	<0.001
* J*_25_ = min(26.97 + 25.76*N*_a_; 105.46 – 59.78 *P*_a_)		**0.30**	**1374.66**		1390.91	<0.001
Savanna (deciduous)						
* J*_25_ = 45.60 + 14.01*N*_a_		0.23	861.59		869.44	<0.001
* J*_25_ = 46.59 + 170.38*P*_a_		0.18	868.26		876.11	<0.001
* J*_25_ = 40.56 + 10.27*N*_a_ + 83.73*P*_a_		0.25	859.78		870.24	<0.001
* J*_25_ = 21.18 + 21.91*N*_a_ + 178.51*P*_a_ – 53.96*N*_a_ *P*_a_		0.26	859.05		872.12	<0.001
* J*_25_ = min(51.28 + 13.06*N*_a_; 29.55 + 337.17*P*_a_)		**0.29**	**855.17**		**869.25**	<0.001
Forest and savanna (deciduous and evergreen)						
* J*_25_ = 38.23 + 19.42*N*_a_		0.30	2431.46		2442.36	<0.001
* J*_25_ = 68.53 + 68.45*P*_a_		0.02	2528.29		2539.19	0.017
* J*_25_ = 40.06 + 20.09*N*_a_ – 26.70*P*_a_		0.30	2432.37		2446.90	<0.001
* J*_25_ = 15.87 + 32.06*N*_a_ + 149.52*P*_a_ – 83.38*N*_a_ *P*_a_		**0.33**	**2421.91**		**2440.09**	<0.001
* J*_25_ = min(29.42 + 24.36*N*_a_; 97.57 + 6.46*P*_a_)		0.33	2422.10		2441.28	<0.001

*N*_a_ nitrogen per unit leaf area (g m^–2^), *P*_a_ phosphorus per unit leaf area (g m^–2^)

Models addressed in detail in the “Results” and “Discussion” sections are highlighted in bold

By contrast, for ${\mathbb{S}}_{\text d}$ it was found that *P*_a_ was nearly as good a predictor as *N*_a_ when considered on its own (*r* ^2^ = 0.18 vs. 0.19) and with the linear model fits including both terms being significantly better than either *N*_a_ or *P*_a_ on their own. Overall, the dual-limitation model was, however, found to be superior to the OLS models according to both the AIC and BIC (*r* ^2^ = 0.30, *p* < 0.001). Although a simple combination of the forest and savanna data suggest that the dual-limitation model is not the best when looking for a common (cross–biome) relationship—in this case it being surpassed by a model containing linear functions of *N*_a_ and *P*_a_ and their interaction term *N*_a_ and *P*_a_—a simple analysis of AIC/BIC and/or the residual sum of squares (RSS) according to a procedure outlined in Lloyd et al. (1989) also shows that this combined (forest + savanna) model provides an inferior fit compared to when forest (i.e. ${\mathbb{F}}_{\text d}$ and ${\mathbb{F}}_{\text e}$ together) and savanna (${\mathbb{S}}_{\text d}$) are considered separately (*p* < 0.001). That is to say, although for the forest species *V*_25_ showed a simple dependency upon *N*_a_, for ${\mathbb{S}}_{\text d}$ an additional role for *P*_a_ is clearly implicated.

A similar picture emerges when models for *J*_25_ are sought with little evidence of a role for *P*_a_ as a modulating factor for either ${\mathbb{F}}_{\text d}$ or ${\mathbb{F}}_{\text e}$ and with *N*_a_ effects apparently dominant for these two forest types (Table 4). As for *V*_25_ there is, however, a clear indication of a role for P for ${\mathbb{S}}_{\text d}$, and with the dual-limitation model giving the best fit. Likewise, when all data are combined, then comparisons of either AIC, BIC or RSS with the individual models show that in any analyses of their *J*_25_ nutrient dependencies, forest and savanna species need to be considered separately.

Fitting separate relationships for both forest and savanna, the resulting goodness of model fit is shown for both *V*_25_ and *J*_25_ in Electronic Supplementary Material, Fig. S3. This shows that in all cases, model predictions involved a much smaller degree of variation than suggested by the observations. An examination of model residuals in terms of the predictor variables *N*_a_ and *P*_a_ along with a range of other potentially confounding covariates such as *M*_a_ and area-based cations (see Electronic Supplementary Material, Figs. S4, S5) did not, however, suggest reasons for concern in terms of any trait-specific systematic bias for either *V*_25_ or *J*_25._ Also note that in Figs. S3 and S4 we have separately identified members of the Fabaceae which, although making up less than 5 % of our data set, are also unusual in their foliar N and P characteristics (Fyllas et al. 2009), especially in relation to photosynthesis (Cernusak et al. 2011). There were, however, no indications from this study that members of this family behaved in any way different to the population sampled as a whole.

**Fig. 5:**
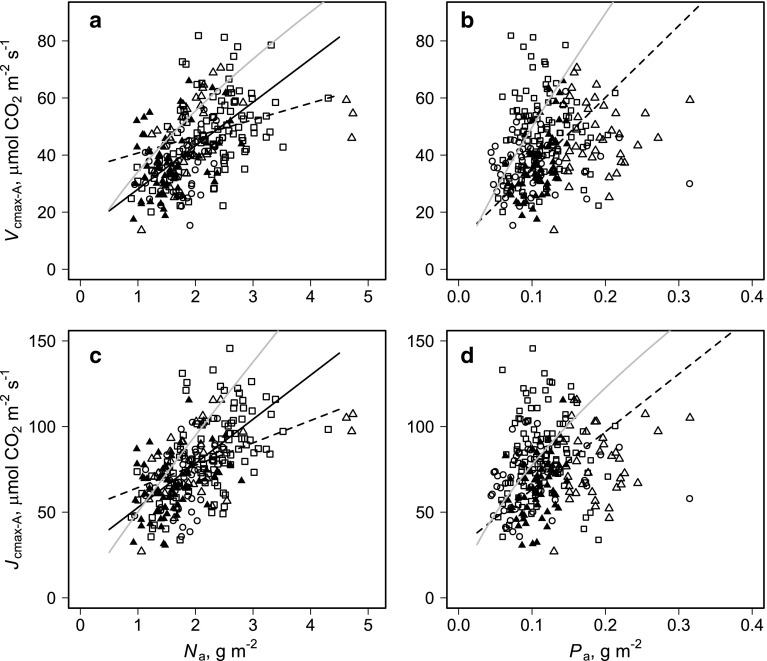
Area-based relationships between **a** estimated Rubisco activity standardized to 25 °C (*V*_25_) and leaf N, **b** *V*_25_ and leaf P, **c** estimated electron transport capacity at 25 °C (*J*_25_) and leaf N, **d** *J*_25_ and leaf P. Deciduous forest (*squares*), evergreen forest (*circles*), savanna (*triangles*). *Filled savanna symbols* show points modelled to be limited by P as per the model fit of Eq. 1 as detailed in Electronic Supplementary Material, Table 1. Also shown from this study are the model fits of Eq. 1 for forest (*full lines*) and savanna (*dotted lines*), along with a previous fit of the same model to a mixture of forest and savanna species sampled along a precipitation transect in West Africa (*grey lines*) as detailed in Domingues et al. (2010)

For both *V*_25_ and *J*_25_ the observed relationships with *N*_a_ and *P*_a_ are shown in Fig. 5. Here, for forest, we have shown the fitted lines for the modelled simple linear *N*_a_ dependencies for both *V*_25_ and *J*_25_ with the dual-limitation model predictions presented only for ${\mathbb{S}}_{\text d}$. This differentiation has been made on the basis of a bootstrapping analysis (Chernick and LaBudde 2011), which showed that for both *V*_25_ and *J*_25_ the (apparently negative) *V*_25_ and *P*_a_ terms were not significantly different from zero (see Table S2 in Electronic Supplementary Material) for both forest types, the implication of this being that *P*_a_ actually exerts no modulating role on the photosynthetic properties of both ${\mathbb{F}}_{\text d}$ and ${\mathbb{F}}_{\text e}$. For *V*_25_ our model clearly suggests that savanna leaves with *N*_a_ < 2.5 g m^−2^ have a higher carboxylation capacity than forest leaves at the same *N*_a_ (Fig. 5a).

Consistent with the simple linear *V*_25_ = f(*N*_a_) model being applied for the forest species, no fitted lines are shown for the forest species for the *P*_a_ relationships of Fig. 5b, d. In both cases, the bootstrapping analysis did, however, show the savanna co-limitation model *P*_a_ slope to be significant with a 0.95 confidence interval for *V*_25_ of 190–375 μmol CO_2 _g^−1^ P s^−1^ with the equivalent range being 246–539 μmol CO_2 _g^−1^ P s^−1^ for *J*_25_ (Electronic Supplementary Material, Table S2).

Also shown for all four parts of Fig. 5 are fitted relationships from the original application of the dual-limitation model of (Domingues et al. 2010) to a wide range of West African tree species and location, including those with a much drier climate (grey lines). In all cases the ‘West African’ slopes are much steeper than found here for Cameroon, where the analysis has been confined to two forest-savanna transition zones. Differences at high *N*_a_ and *P*_a_ are particularly marked.

## Discussion

Using as our individual unit of variation the individual leaf (rather than the tree or species), we found unequivocal support for our first hypothesis viz. that the scaling between foliar N and P and the nature of the photosynthesis-nutrient relationship would be different for forest and savanna species. This can be seen from Fig. 4d where at any given leaf N (area basis) savanna trees had significantly higher P than their forest counterparts and Fig. 5 where the equations of Tables 2 and 3 clearly shown different relationships of both *V*_c(max)_ and *J*_max_ with both N and P for forest vs. savanna species.

On the other hand, although we had also hypothesized that savanna trees should show more indications of being limited by N, if anything the opposite was the case. This is because the best-fitting relationship between the photosynthetic parameters *V*_c(max)_ and *J*_max_ for both evergreen and deciduous forest trees were a simple linear relationship with *N*_a_ with no relationship at all when a linear model with P content was tested. By contrast, the savanna species (all of which were deciduous) showed significant relationships with *P*_a_ as well as with *N*_a_—albeit with a different N dependence to that found for the forest species. Thus, if anything, the indications were for a greater limitation by P on photosynthesis in savanna as opposed to forest trees.

Biome history may be important in explaining these results. For example any forest refugia in Africa at the Last Glacial Maximum (Anhuf et al. 2006) would have been most likely to have occurred where both precipitation regime and soil conditions remained most favourable for forest tree function. So, with deeper tropical soils of a high water holding capacity also typically being of a low P status due to their long history of extreme weathering (Quesada et al. 2010), specific adaptions to a chronically low *P*_a_ for forest trees seem likely, for example in the replacement of phospholipids by galactolipids and sulpholipids under condition of low P supply (Lambers et al. 2012; Tjellström et al. 2008; Zhang et al. 2014).

With leaves intercepting light on a per unit area basis and spurious correlations possible when two unrelated area-based entities are transformed to a mass basis—the so called ‘lulu effect’ (Lloyd et al. 2013)-we saw for the current paper no practical reason to analyse our photosynthesis-nutrient dependencies on per unit mass basis (see also Osnas et al. 2013). Nevertheless, for the purposes of illustration our area-based analysis is repeated on a mass basis as part of the Electronic Supplementary Material (Tables S3, S4). This shows—in addition to the inevitable higher correlations associated with a ‘common-element’ correlation (Lloyd et al. 2013)—that for the forest species it is more often than not the more complex models involving not only both *N*_m_ and *P*_m_, but also their interaction, that have the lowest AIC and/or BIC. This is as opposed to the simple forest species linear N dependency for both *V*_25_ and *J*_25_ for the area-based fits. Overall these results are consistent with the assertion that in multivariate cases a simple area-to-mass conversion can easily give rise to variables not actually associated with the dependent variable appearing to be functionally linked (Lloyd et al. 2013). We also note that whilst the area-based models showed little bias in their residuals when examined as a function of *M*_a_, *N*:*P* and a range of area based leaf-nutrient measures (Figs S3, S4), this was not the case for the mass-based models for which there was a bias towards positive residuals at low *M*_a_ (Figs S5, S6). The lack of any obvious dependency of the model-fit residuals on leaf cation concentrations, area-based leaf S or leaf *N*:*P* ratios suggests that—at least in our case—there is no need to invoke additional factors such as variations in leaf K (Battie-Laclau et al. 2014) into our dual-dependency N-P based model. Though that is not to say, of course, that such elements might not have important roles in influencing tropical vegetation structure and function independent of the photosynthetic process (Schrodt et al. 2014; Veenendaal et al. 2014).

With our earlier analyses using the formulation of Eq. 1 having actually focussed on mass-based model fits (Domingues et al. 2010), there does, however, remain the question: to what extent are some previous conclusions of Domingues et al. (2010) regarding the relative roles on N and P still valid? The answer is that, with only minor modifications, they still hold. For example, in that paper we also showed that area-based fits of the dual-limitation model implied a role for both N and P as alternate limiting factors for photosynthesis (in addition to the mass-based models) and with area-based comparisons with simple linear models also showing the min–min model to have the lowest AIC. Indeed, the analysis here should be best considered a refinement of the work of Domingues et al. (2010), probing further into the nature of the apparent different nutrient/photosynthesis relationships identified for species associated with the different rainfall environments first identified there.

It is unlikely that the differences between forest and savanna in their photosynthesis–N relations (Figs. 4a, 5a, c) were simply a consequence of the sampled forest tree leaves being from a lower light environment. This is because considerable effort was put into ensuring that leaves from both vegetation formation types were sampled only from upper-canopy sun-exposed environments (See “Materials and methods”). Rather, and especially as broadly similar results have also been reported for a forest-savanna comparison in tropical northern Australia (Bloomfield et al. 2014), this difference between the two vegetation formation types in their photosynthesis–nutrient relationships seems something more fundamental. Both Domingues et al. (2010) and (Bloomfield et al. 2014) discuss at some length possible reasons for forest trees having an apparently less efficient use of N, focussing on the idea of an increased allocation of N to non-photosynthetic compounds when conditions favouring a longer leaf longevity are also combined with a more variable light environment.

The question remains, however, as to the extent of the validity of the original parameterisation of Domingues et al. (2010), or the new forest parameterisation developed here, when applied to tropical forest trees growing on low-P availability soils, such as those which cover much of the eastern Amazon Basin (Quesada et al. 2011), especially as already investigated as part of the modelling studies of Mercado et al. (2011) and Fyllas et al. (2014). Trees on such soils do, nevertheless, typically have a foliage of a much lower *P*_a_ than encountered here (Table 2), so it will only be with further dedicated measurements under the full spectrum of *N*:*P* variability and across a range of different growth forms that we will be able to ascertain the generality (or most likely otherwise) of any photosynthesis–nutrient relationships developed.

Most likely the dual-limitation model applies because for some specific locations and/or for some particular times of the year, P is rate limiting, whilst for other times/places it is N which constrains photosynthetic productivity. As for the Amazon Basin forest case discussed above, these regional variations, arising mostly from soil variations—but also clearly depending on vegetation formation type—will give rise to variations in the rates of photosynthesis, these linking to variations in stand-level productivity (Fyllas et al. 2014; Mercado et al. 2011) and thus presumably important when parameterizing global vegetation models (e.g. Sitch et al. 2008 and Piao et al. 2013). This emphasizes a need for the development of new realistic models of ecosystem N and P cycling that include soil biogeochemical processes in a realistic manner (Fisher et al. 2010; Goll et al. 2012; Ostle et al. 2009; Thomas et al. 2013; Xu et al. 2012; Yang et al. 2013). But whatever the case, the results presented here along with those of other recent studies (Bloomfield et al. 2014; Domingues et al. 2010; Rossatto et al. 2013) clearly indicate that no single unifying woody tropical vegetation photosynthesis–nutrient relationship is likely to be found.

### **Author contribution statement**

TFD; collected and analyzed data, wrote manuscript, prepare figures, FYI; collected data, field support TF; collected data, field support, JG; institutional support, discussion of results, manuscript structure, GS; collected data, field support, OS; botanical identification, field support, FS; collected data, field support, BS; logistical support, botanical identification HT; botanical identification, field support, EV; discussion of results, manuscript structure SL; logistical support, site selection, field activities planning, JL; Project coordinator, manuscript writing, discussion of results.

## Electronic supplementary material

Below is the link to the electronic supplementary material.

Supplementary material 1 (DOCX 1572 kb)
