# Supplementary material for: Biome-specific effects of nitrogen and phosphorus on the photosynthetic characteristics of trees at a forest-savanna boundary in Cameroon
Source: Oecologia. 2015 Mar 10;178(3):659–72. doi: 10.1007/s00442-015-3250-5 (PMC4472954; doi:10.1007/s00442-015-3250-5)
Supplement: Supplementary file 1 — Supplementary material 1 (DOCX 1572 kb) [file 442_2015_3250_MOESM1_ESM.docx]

**Supplementary information to accompany**

**Biome-specific effects of nitrogen and phosphorus on the photosynthetic characteristics of trees at a forest-savanna boundary in Cameroon**

*by* T. F. Domingues, P. Meir, J. Grace, E. M. Veenendaal, F.Y. Ishida, B. Sonké, T. R. Feldpausch, G. Saiz, F. I. Schrodt, H. Taedoumg, O. Sene, S. L. Lewis & Jon Lloyd.

**CONTENTS____________________________________________________________**

Part 1: Site and sampling information

Part 2: Effects of internal conductance assumptions of estimates of *V*_max_ and *J*_max_

Part 3: Evaluations of min-min model fit (including residual plots)

Part 4. Mass-based model output and residual plots

________________________________________________________________________

**Part 1: Site and sampling details**


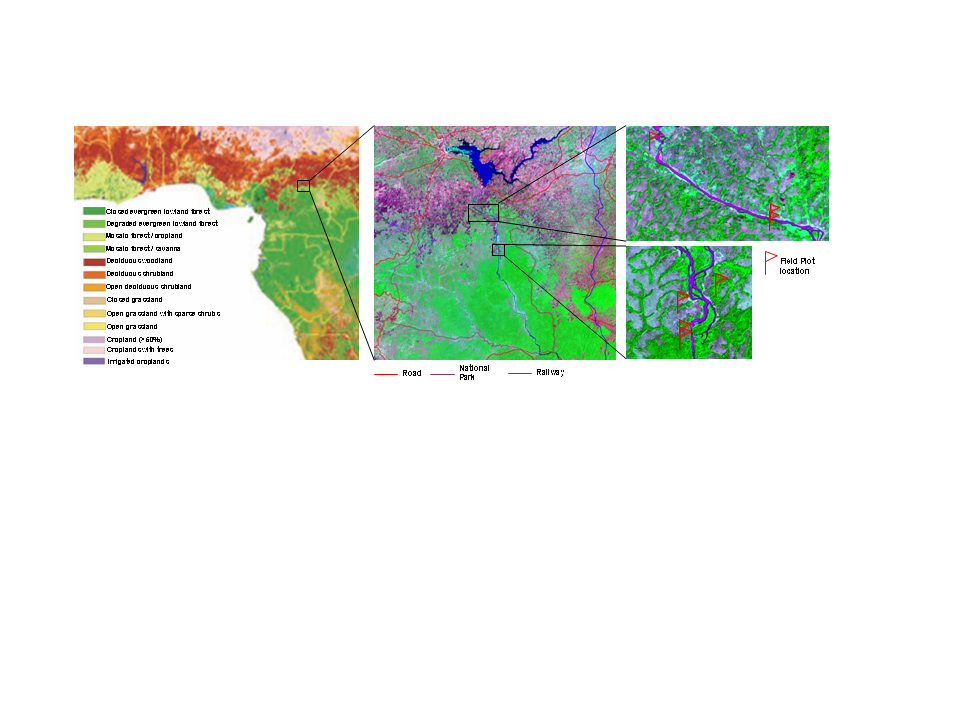


**Fig. S1** A section of a vegetation map of Africa taken from [Mayaux et al. (2004)](http://www.sciencedirect.com/science/article/pii/S0034425711001337#bb0220), showing the location of the study area within Cameroon (left panel) with field plot locations within the forest/savanna mosaic shown (centre and right hand panels). The latter come from the satellite imagery analysis of Mitchard et al. (2009) for which forest areas are typically green with savanna areas blue.

Table S1: List of species sampled at the Mbam-Djerem National Park, sample size, and species averages ± standard deviation of leaf parameters: Mass to area ratio (*M*_A_), area based photosynthetic rate at saturating light and ambient CO_2_ (*A*_Sat_), stomatal conductance measured at *A*_Sat_ (*g*_s_@*A*_Sat_), internal conductance (*g*_i_), mass based maximum carboxylation capacity at 25°C (*V*_cmax-DW_), mass based maximum electron transport capacity at 25°C (*J*_max-DW_), mass based leaf concentrations of nitrogen (N_DW_), phosphorus (P_DW_), and sulphur (S_DW_), and the stable carbon (δ^13^C) and nitrogen (δ^15^N) isotopic ratio.

| **Plot** | **Species** | **# of**  **individuals**  **(# of leaves)** | ***M*_A_**  **g m^-2^** | ***A*_Sat_**  **μmol m^-2^ s^-1^** | ***g*_s_@*A*_Sat_**  **mol m^-2^ s^-1^** | ***g*_i_**  **μmol m^-2^ s^-1^Pa^-1^** | ***V*_cmax-DW_**  **μmol g^-1^ s^-1^** | ***J*_max-DW_**  **μmol g^-1^ s^-1^** | **N_DW_**  **mg g^-1^** | **P_DW_**  **mg g^-1^** | **δ^13^C**  **‰** | **δ ^15^N**  **‰** |
| --- | --- | --- | --- | --- | --- | --- | --- | --- | --- | --- | --- | --- |
| MDJ-1 | Not identified | 1 (2) | 64.6 | 6.0 ± 1.7 | 0.12 ± 0.05 | 4.6 ± 2.6 | 0.40 | 0.74 | 29.6 | 0.94 | -31.1 | 4.0 |
| MDJ-1 | *Berlinia grandiflora* | 1 (2) | 100.9 ± 2.3 | 9.5 ± 1.4 | 0.09 ± 0.02 | 14.4 ± 0.1 | 0.51 ± 0.00 | 0.87 ± 0.04 | 24.0 ± 1.3 | 1.58 ± 0.88 | -28.7 ± 0.1 | 2.5 ± 0.3 |
| MDJ-1 | *Celtis adolfi-fridericii* | 1 (3) | 106.1 ± 10.3 | 8.9 ± 1.6 | 0.15 ± 0.06 | 6.5 ± 6.4 | 0.39 ± 0.09 | 0.75 ± 0.17 | 17.2 ± 2 | 1.14 ± 0.08 | -28.6 ± 0.2 | 2.6 ± 0.3 |
| MDJ-1 | *Cola lateritia* | 1 (3) | 78.6 ± 0.3 | 4.4 ± 1.3 | 0.11 ± 0.02 | 14.7 ± 0.1 | 0.19 ± 0.10 | 0.35 ± 0.24 | 16.3 ± 0.1 | 1.11 ± 0.08 | -31.0 ± 0.4 | 1.4 ± 0.2 |
| MDJ-1 | *Ficus exasperata* | 1 (4) | 114.9 ± 10.2 | 13.5 ± 4.1 | 0.37 ± 0.16 | 11.0 ± 7.4 | 0.51 ± 0.03 | 0.85 ± 0.07 | 22.3 ± 1 | 1.22 ± 0.19 | -29.4 ± 0.3 | 1.3 ± 1.0 |
| MDJ-1 | *Ficus variifolia* | 1 (4) | 39.0 ± 1.3 | 8.3 ± 0.8 | 0.24 ± 0.06 | 4.3 ± 7.0 | 0.80 ± 0.07 | 1.42 ± 0.20 | 25.2 ± 2.5 | 1.6 ± 0.24 | -31.6 ± 0.2 | 3.8 ± 0.8 |
| MDJ-1 | Fabaceae | 1 (4) | 87.0 ± 14.0 | 10.4 ± 0.9 | 0.15 ± 0.04 | 1.5 ± 1.2 | 0.68 ± 0.05 | 1.23 ± 0.07 | 32.8 ± 1.1 | 1.57 ± 0.33 | -28.3 ± 0.5 | 1.2 ± 0.1 |
| MDJ-1 | *Markhamia lutea* | 3 (9) | 52.7 ± 23.3 | 10 ± 6.1 | 0.19 ± 0.15 | 4.5 ± 6.4 | 1.34 ± 0.51 | 2.32 ± 0.57 | 33.0 ± 8.8 | 2.38 ± 0.97 | -30.7 ± 1.3 | 2.5 ± 0.3 |
| MDJ-1 | *Milicia excelsa* | 1 (5) | 123.7 ± 12.5 | 11.8 ± 3.7 | 0.19 ± 0.09 | 1.5 ± 1.5 | 0.47 ± 0.05 | 0.74 ± 0.12 | 18.5 ± 0.3 | 1.35 ± 0.33 | -27.6 ± 0.3 | 0.0 ± 0.5 |
| MDJ-1 | *Pouteria alnifolia* | 1 (4) | 87.4 ± 12.1 | 10.4 ± 1.1 | 0.18 ± 0.04 | 1.9 ± 1.8 | 0.55 ± 0.12 | 0.98 ± 0.14 | 29.2 ± 1.4 | 1.37 ± 0.08 | -30.4 ± 0.2 | 4.1 ± 0.2 |
| MDJ-1 | *Tabernaemontana crassa* | 1 (4) | 64.8 ± 34.5 | 7.4 ± 1.7 | 0.10 ± 0.03 | 10.9 ± 7.3 | 0.38 ± 0.39 | 0.84 ± 0.80 | 29.6 ± 1.4 | 4.4 ± 0.44 | -30.8 ± 0.2 | 4.2 ± 0.8 |
| MDJ-1 | *Trichilia rubescens* | 1 (2) | 84.9 | 7.4 ± 2.8 | 0.18 ± 0.08 | 14.1 ± 0.2 | 0.21 | 0.48 | 25.4 ± 4.2 | 1.09 ± 0.32 | -29.4 ± 0.2 | 3.5 ± 2.9 |
| MDJ-1 | *Trilepisium madagascariense* | 2 (8) | 98.6 ± 12.3 | 11 ± 1 | 0.15 ± 0.04 | 9.4 ± 1.7 | 0.53 ± 0.04 | 0.79 ± 0.04 | 22.5 ± 0.5 | 1.49 ± 0.23 | -29.7 ± 0.6 | 2.3 ± 0.9 |
| MDJ-1 | *Voacanga* sp. | 1 (3) | 26.4 ± 0.9 | 5.7 | 0.05 | 4.9 ± 8.4 | 1.52 | 3.04 | 32.8 ± 15.7 | 2.69 ± 1.54 | -30.6 ± 0.5 | 1.1 ± 0.1 |
| MDJ-2 | *Annona senegalensis* | 1 (4) | 153.4 ± 7.2 | 19 ± 1.3 | 0.60 ± 0.06 | 7.8 ± 6.8 | 0.46 ± 0.06 | 0.67 ± 0.09 | 15.9 ± 0.2 | 0.93 ± 0.1 | -30.2 ± 0.3 | 2.4 ± 0.1 |
| MDJ-2 | *Crossopteryx febrifuga* | 2 (4) | 108 ± 13.9 | 11 ± 2.4 | 0.34 ± 0.01 | 5.2 ± 4.6 | 0.39 ± 0.03 | 0.61 ± 0.05 | 14.7 ± 1.1 | 1.09 ± 0.37 | -30.3 ± 0.8 | -0.4 ± 0.6 |
| MDJ-2 | *Daniellia oliveri* | 1 (1) | 115.6 |  |  | 11.8 | 0.19 | 0.31 | 21.7 | 0.90 | -28.3 | 0.6 |
| MDJ-2 | *Piliostigma thonningii* | 1 (4) | 125 ± 6.6 | 14.9 ± 4.2 | 0.29 ± 0.06 | 9.7 ± 6.4 | 0.43 ± 0.01 | 0.63 ± 0.02 | 16.1 ± 1.0 | 1.02 ± 0.23 | -30.4 ± 0.2 | -0.7 ± 0.3 |
| MDJ-2 | *Ximenia americana* | 1 (4) | 160.2 ± 8.7 | 14.4 ± 1.7 | 0.32 ± 0.06 | 4.6 ± 6.3 | 0.39 ± 0.02 | 0.67 ± 0.05 | 29.3 ± 1.5 | 1.69 ± 0.31 | -30.9 ± 0.2 | -0.8 ± 0.1 |
| MDJ-3 | *Amphimas pterocarpoides* | 1 (1) | 67.7 | 7.4 | 0.09 | 1.6 | 0.61 | 1.2 | 37.1 | 1.20 | -28.4 | 3 |
| MDJ-3 | *Antidesma laciniatum* | 1 (4) | 69.4 ± 6.7 | 12.8 ± 1.7 | 0.21 ± 0.08 | 10.2 ± 6.8 | 0.71 ± 0.04 | 1.13 ± 0.05 | 24.0 ± 1.5 | 0.99 ± 0.16 | -30.6 ± 0.4 | 2.9 ± 0.2 |
| MDJ-3 | *Funtumia elastica* | 1 (3) | 104.8 ± 5.7 | 7 ± 3.1 | 0.08 ± 0.07 | 5.6 ± 6.3 | 0.48 ± 0.13 | 0.74 ± 0.17 | 22.6 ± 1.5 | 0.96 ± 0.16 | -30.5 ± 0.5 | 1.7 ± 0.4 |
| MDJ-3 | *Hannoa* sp. | 1 (4) | 119.5 ± 8.8 | 11.7 ± 2.3 | 0.16 ± 0.04 | 4.4 ± 7.1 | 0.49 ± 0.06 | 0.83 ± 0.06 | 28.1 ± 1.5 | 1.36 ± 0.30 | -29.2 ± 0.4 | 3.5 ± 0.1 |
| MDJ-3 | *Homalium* sp. | 1 (4) | 137.1 ± 9.3 | 12.3 ± 1.6 | 0.24 ± 0.06 | 8.1 ± 8.7 | 0.38 ± 0.03 | 0.56 ± 0.04 | 17.4 ± 0.5 | 0.52 ± 0.05 | -31.7 ± 0.6 | 2.7 ± 0.3 |
| MDJ-3 | Fabaceae | 3 (11) | 90.3 ± 14.9 | 13.1 ± 3.8 | 0.29 ± 0.12 | 6.0 ± 5.4 | 0.79 ± 0.22 | 1.43 ± 0.4 | 29.7 ± 1.7 | 1.40 ± 0.72 | -29.5 ± 0.6 | 2.5 ± 1.5 |
| MDJ-3 | *Maprounea membranacea* | 2 (4) | 66.5 ± 39.0 | 5.9 ± 1.5 | 0.08 ± 0.02 | 5.1 ± 6.7 | 0.70 ± 0.29 | 1.06 ± 0.37 | 22.0 ± 3.2 | 1.04 ± 0.01 | -30.8 ± 0.2 | 2.2 ± 0.5 |
| MDJ-3 | *Milicia excelsa* | 1 (4) | 132.5 ± 8.1 | 16.7 ± 3.8 | 0.33 ± 0.03 | 6.1 ± 7.1 | 0.48 ± 0.07 | 0.71 ± 0.05 | 22.7 ± 1.0 | 1.03 ± 0.17 | -28.4 ± 0.3 | 3.3 ± 0.4 |
| MDJ-3 | *Oncoba glauca* | 1 (1) | 69.6 | 4.2 | 0.04 | 6.9 ± 7.7 | 0.29 | 0.51 | 17.7 | 0.86 | -31.4 | 2.5 |
| MDJ-3 | *Oncoba* sp. | 1 (4) | 108.9 ± 6.0 | 7 ± 3.0 | 0.09 ± 0.05 | 4.4 ± 6.2 | 0.31 ± 0.03 | 0.67 ± 0.09 | 15.5 ± 1.2 | 0.88 ± 0.05 | -30.8 ± 0.6 | 2.8 ± 0.3 |
| MDJ-3 | *Sapium* sp. | 2 (5) | 104.3 ± 20.7 | 9.5 ± 4.5 | 0.18 ± 0.14 | 2.2 ± 1.1 | 0.50 ± 0.16 | 0.88 ± 0.14 | 21.6 ± 0.2 | 1.14 ± 0.08 | -30 ± 3.4 | 2.7 ± 1.0 |
| MDJ-3 | *Spondias* sp. | 1 (3) | 75.7 ± 6.3 | 10.2 ± 4.5 | 0.20 ± 0.11 | 0.9 ± 0.4 | 0.71 ± 0.18 | 1.04 ± 0.27 | 23.3 ± 1.2 | 1.44 ± 0.26 | -29.5 ± 0.3 | 2.4 ± 0.5 |
| MDJ-3 | *Trilepisium madagascariense* | 1 (4) | 122.8 ± 3.0 | 11.3 ± 1.3 | 0.15 ± 0.06 | 5.8 ± 6.8 | 0.44 ± 0.06 | 0.68 ± 0.05 | 25.3 ± 0.8 | 1.09 ± 0.11 | -29.3 ± 0.9 | 2.3 ± 0.8 |
| MDJ-4 | *Crossopteryx febrifuga* | 3 (12) | 107.8 ± 11.1 | 7.9 ± 2.7 | 0.21 ± 0.09 | 6.2 ± 1.7 | 0.32 ± 0.13 | 0.51 ± 0.19 | 10.6 ± 0.8 | 1.06 ± 0.12 | -30 ± 0.5 | 0.4 ± 0.2 |
| MDJ-4 | *Hymenocardia acida* | 3 (10) | 120.1 ± 11.2 | 14.8 ± 3.6 | 0.37 ± 0.12 | 9.9 ± 3.2 | 0.45 ± 0.15 | 0.70 ± 0.23 | 14.7 ± 0.9 | 1.45 ± 0.19 | -30.7 ± 1.0 | -0.1 ± 0.9 |
| MDJ-4 | *Maprounea membranacea* | 3 (9) | 112.3 ± 12.8 | 9 ± 1.3 | 0.22 ± 0.03 | 2.7 ± 2.5 | 0.32 ± 0.07 | 0.57 ± 0.13 | 12.5 ± 1.3 | 0.63 ± 0.19 | -30.5 ± 0.6 | 0.6 ± 0.4 |
| MDJ-4 | *Syzygium guineense* | 3 (11) | 165.4 ± 16.6 | 13.5 ± 2.1 | 0.35 ± 0.08 | 9.3 ± 3.2 | 0.30 ± 0.07 | 0.47 ± 0.10 | 9.9 ± 0.4 | 0.88 ± 0.13 | -30.1 ± 0.5 | -0.6 ± 2.3 |
| MDJ-5 | *Combretum* sp. | 3 (9) | 103.2 ± 7.5 | 10 ± 0.7 | 0.24 ± 0.05 | 6.9 ± 2.3 | 0.41 ± 0.06 | 0.66 ± 0.07 | 15.9 ± 1.7 | 0.97 ± 0.12 | -30.4 ± 0.8 | -0.1 ± 0.3 |
| MDJ-5 | *Funtumia elastica* | 4 (8) | 76.7 ± 12.3 | 12 ± 1.4 | 0.30 ± 0.08 | 6.2 ± 3.6 | 0.68 ± 0.05 | 1.10 ± 0.11 | 26.2 ± 2.4 | 1.17 ± 0.06 | -30.5 ± 0.8 | 0.1 ± 1.1 |
| MDJ-5 | Ochnaceae | 3 (10) | 90.7 ± 14.3 | 13 ± 3.4 | 0.32 ± 0.14 | 7.1 ± 2.9 | 0.56 ± 0.05 | 0.93 ± 0.04 | 22.5 ± 1.2 | 0.79 ± 0.08 | -29.5 ± 0.6 | 1.9 ± 0.9 |
| MDJ-5 | *Spondias mombin* | 3 (9) | 87.6 ± 15.4 | 8.8 ± 1.4 | 0.25 ± 0.10 | 4.0 ± 5.3 | 0.43 ± 0.10 | 0.65 ± 0.11 | 17.3 ± 2.0 | 1.02 ± 0.07 | -30.4 ± 0.6 | 1.6 ± 1.1 |
| MDJ-5 | *Vitex doniana* | 2 (5) | 192.3 ± 46.3 | 13.3 ± 2.3 | 0.43 ± 0.06 | 8.5 ± 1.4 | 0.31 ± 0.00 | 0.49 ± 0.07 | 13.3 ± 2.7 | 0.80 ± 0.15 | -28.2 ± 1.9 | 1.4 ± 0.7 |
| MDJ-5 | *Xylopia aethiopica* | 3 (8) | 138.6 ± 8.7 | 12.6 ± 2.2 | 0.18 ± 0.06 | 4.6 ± 2.9 | 0.48 ± 0.04 | 0.80 ± 0.08 | 21.7 ± 1.5 | 0.69 ± 0.05 | -30.6 ± 0.8 | 1.2 ± 0.6 |
| MDJ-7 | Not identified | 1 (3) | 72.0 ± 10.1 | 13 ± 0.9 | 0.25 ± 0.05 | 1.3 ± 0.3 | 0.99 ± 0.22 | 1.46 ± 0.33 | 31.3 ± 3.6 | 1.11 ± 0.29 | -32.5 ± 0.2 | 1.4 ± 0.7 |
| MDJ-7 | *Homalium letestui* | 1 (1) | 170.1 |  |  |  |  |  | 18.4 | 0.59 | -29.5 | 3.5 |
| MDJ-7 | *Irvingia gabonensis* | 1 (3) | 100.5 ± 7.9 | 5.8 ± 0.5 | 0.08 ± 0.00 | 4.7 ± 7.6 | 0.35 ± 0.12 | 0.56 ± 0.25 | 19.3 ± 0.5 | 0.65 ± 0.09 | -29.8 ± 0.3 | 5.2 ± 0.1 |
| MDJ-7 | *Maranthes chrysophylla* | 1 (3) | 116.1 ± 4.5 | 9.4 ± 2.2 | 0.31 ± 0.05 | 0.5 ± 0.1 | 0.46 ± 0.15 | 0.78 ± 0.15 | 16.2 ± 0.8 | 0.68 ± 0.18 | -31.9 ± 0.2 | 2.7 ± 0.2 |
| MDJ-7 | *Milicia excelsa* | 1 (3) | 109.1 ± 1.6 | 17.8 ± 1.8 | 0.45 ± 0.15 | 2.7 ± 2.6 | 0.69 ± 0.05 | 0.96 ± 0.08 | 23.7 ± 0.9 | 0.98 ± 0.12 | -29.8 ± 0.9 | 2.1 ± 0.3 |
| MDJ-7 | *Oncoba glauca* | 2 (6) | 86.7 ± 10.1 | 8.5 ± 1.5 | 0.13 ± 0.01 | 9.2 ± 1.1 | 0.51 ± 0.04 | 0.92 ± 0.03 | 19.1 ± 0.6 | 0.92 ± 0.09 | -31.8 ± 0.8 | 3.8 ± 0.3 |
| MDJ-7 | *Parkia biglobosa* | 2 (5) | 102.5 ± 2.4 | 10.6 ± 0.8 | 0.17 ± 0.03 | 4.2 ± 5.9 | 0.48 ± 0.10 | 0.72 ± 0.13 | 27.3 ± 3.8 | 1.24 ± 0.16 | -29.7 ± 1.0 | 3.9 ± 0.5 |
| MDJ-7 | *Pterygota bequaertii* | 1 (3) | 92.1 ± 12.6 | 11.4 ± 3.2 | 0.04 ± 0.00 | 4.9 ± 8.5 | 0.59 | 1.20 | 27.5 ± 0.4 | 1.39 ± 0.20 | -28.3 ± 0.5 | 3.8 ± 0.2 |
| MDJ-7 | *Pycnanthus angolensis* | 1 (3) | 142.9 ± 48.1 | 4.4 | 0.05 | 0.2 ± 0.3 |  |  | 18.5 ± 1.3 | 1.14 ± 0.23 | -28.5 ± 0.1 | 4.9 ± 0.1 |
| MDJ-7 | *Spondias mombin* | 1 (3) | 86.2 ± 4.9 | 5.1 ± 2.9 | 0.06 ± 0.03 | 3.9 ± 5.4 | 0.41 ± 0.18 | 0.62 ± 0.25 | 20.3 ± 0.2 | 1.15 ± 0.13 | -29.9 ± 0.5 | 2.8 ± 0.3 |
| MDJ-7 | *Syzygium guineense* | 1 (3) | 132.4 ± 12.8 | 4.6 ± 3.8 | 0.21 ± 0.06 | 4.6 ± 7.6 | 0.22 ± 0.13 | 0.58 ± 0.15 | 13.7 ± 0.6 | 0.48 ± 0.05 | -30.9 ± 0.2 | 2.2 ± 0.4 |
| MDJ-7 | *Trichilia rubescens* | 1 (2) | 70.2 ± 0.5 | 10.8 ± 0.5 | 0.19 ± 0.01 | 2.0 ± 1.3 | 0.71 ± 0.07 | 1.10 ± 0.03 | 26.8 ± 1.8 | 1.44 ± 0.27 | -30.3 ± 0.3 | 2.6 ± 1.0 |
| MDJ-7 | *Uapaca guineensis* | 1 (1) | 95.8 | 11.5 | 0.06 | 1.1 | 0.75 | 1.09 | 18.3 | 0.76 | -31.4 | 4.0 |
| MDJ-7 | *Xylopia aethiopica* | 1 (3) | 144.3 ± 16.3 | 6.1 ± 3.5 | 0.25 ± 0.04 | 3.3 ± 3.7 | 0.30 ± 0.05 | 0.68 ± 0.15 | 19.0 ± 0.9 | 0.75 ± 0.06 | -30.8 ± 0.2 | 2.5 ± 0.5 |
| MDJ-8 | *Crossopteryx febrifuga* | 3 (9) | 117.6 ± 13.4 | 5.3 ± 1.4 | 0.11 ± 0.03 | 5.1 ± 2.9 | 0.20 ± 0.05 | 0.33 ± 0.08 | 8.6 ± 0.9 | 1.73 ± 0.39 | -29.3 ± 1.2 | -2.0 ± 0.2 |
| MDJ-8 | *Daniellia oliveri* | 3 (10) | 144.9 ± 12.7 | 4.7 ± 2.1 | 0.11 ± 0.04 | 6.8 ± 4.9 | 0.11 ± 0.09 | 0.20 ± 0.12 | 9.3 ± 1.5 | 0.58 ± 0.03 | -28.5 ± 0.6 | -0.7 ± 0.7 |
| MDJ-8 | *Maytenus senegalensis* | 3 (9) | 195 ± 17.4 | 6.4 ± 2.4 | 0.12 ± 0.04 | 0.6 ± 0.3 | 0.15 ± 0.03 | 0.27 ± 0.04 | 5.4 ± 0.4 | 2.09 ± 1.22 | -29.1 ± 0.2 | -0.9 ± 0.1 |
| MDJ-8 | *Piliostigma thonningii* | 3 (11) | 145.6 ± 8 | 10.9 ± 4.1 | 0.22 ± 0.08 | 11.4 ± 1.7 | 0.25 ± 0.12 | 0.39 ± 0.18 | 10.0 ± 0.8 | 2.26 ± 0.36 | -29.6 ± 0.4 | -1.6 ± 0.2 |

**Part 2: Effects of internal conductance assumptions of estimates of *V*_max_ and *J*_max_**

**Fig. S2.** Relationship between photosynthetic parameters derived from fitting a biochemical photosynthesis model (Faquhar et al, 1980) without considering a mesophyll conductance (*g*_m_) (CO_2_ concentration at the chloroplast [*C*_i_] equal to that at the mesophyll [*C*_c_]) and considering a finite *g*_m_ (*C*_c_ less than *C*_i_).

**Part 3: Evaluations of dual-limitation model and parameter fits**

**Fig. S2** Model predictions of estimated Rubisco activity standardised to 25 °C (*V*_25_*)and (b)* estimated electron transport capacity @ 25 °C according to Eq. 2.

**Fig. S3** *V*_max_: Plots of residuals versus observed values of leaf mass per unit area (*M*_a_); area based nutrients and foliar N:P ratios. Members of the Fabaceae family: closed squares; all other species: open circles.

**Fig. S4** *J*_max_: Plots of residuals versus observed values of leaf mass per unit area (*M*_a_); area based nutrients and foliar N:P ratios. Members of the Fabaceae family: closed squares; all other species: open circles.

| **Equation** | **Functional Group** | **Coef. *a*** | **Coef. *b*** | **Coef. *c*** | **Coef. *d*** |
| --- | --- | --- | --- | --- | --- |
| *V* _25_ | *F*_d_ | -12.19 – 24.34 | 10.1 – 34.61 | 51.26 – 85.58 | -224.15 – 9.86 |
| *V* _25_ | *F*_e_ | 5.24 – 26.5 | 5.76 – 18.07 | 40.7 – 67.62 | 21.62 – 64.29 |
| *V* _25_ | *F*_d_ + *F*_e_ | 5.55 – 19.57 | 11.6 – 19.36 | 51.81 – 85.11 | -176.86 – 7.01 |
| *V* _25_ | *S*_d_ | 14.7 – 44.57 | 2.05 – 17.07 | -3.07 – 17.31 | 190.65 – 375.08 |
|  |  |  |  |  |  |
| *J* _25_ | *F*_d_ | -16.11 – 47.72 | 16.62 – 58.27 | 81.87 – 163.76 | -496.35 – 207.43 |
| *J* _25_ | *F*_e_ | 16.11 – 48.46 | 11.67 – 30.18 | 72.05 – 107.03 | 56.99 – 125.03 |
| *J* _25_ | *F*_d_ + *F*_e_ | 14.46 – 39.98 | 18.99 – 32.69 | 81.02 – 145.08 | -285.05 – 217.54 |
| *J* _25_ | *S*_d_ | 29.67 – 64.83 | 7.91 – 26.11 | 7.39 – 43.56 | 246.36 – 538.86 |

**Table S2** Confidence intervals for the regression coefficients of the min-min model (Eq. 2). Functional groups as in Table 3. Ranges were created by bootstrap approach (2.5 and 97.5 % quantiles from 10.000 trials, random sampling with replacement).

**Part 3. Mass-based model output and residual diagnostics**

| **Equation** | ***r*^2^** | ***AIC*** | | ***BIC*** | ***p*** |
| --- | --- | --- | --- | --- | --- |
| Forest Evergreen | | | | | |
| *V*_c(max),m_ = – 0.005 + 0.022*N*_m_ | **0.53** | **-53.46** | | **-47.67** | <0.001 |
| *V*_c(max),m_ = 0.359 + 0.097*P*_m_ | 0.20 | -26.25 | | -20.45 | 0.001 |
| *V*_c(max),m_ = – 0.008 + 0.022*N*_m_ – 0.004*P*_m_ | 0.52 | -51.49 | | -43.76 | <0.001 |
| *V*_c(max),m_ = 0.142 + 0.017*N*_m_ – 0.154*P*_m_ + 0.005*N*_m_ *P*_m_ | 0.54 | -52.27 | | -42.62 | <0.001 |
| *V*_c(max),m_ = min(-0.048 + 0.024*N*_m_; 0.378 + 0.157*P*_m_) | 0.54 | -52.36 | | -41.70 | <0.001 |
| Forest Deciduous | | | | | |
| *V*_c(max),m_ = – 0.107 + 0.026*N*_m_ | 0.46 | -63.26 | -55.29 | | <0.001 |
| *V*_c(max),m_ = 0.202 + 0.254*P*_m_ | 0.33 | -40.92 | -32.96 | | <0.001 |
| *V*_c(max),m_ = – 0.110 + 0.020*N*_m_ + 0.124*P*_m_ | 0.51 | -72.28 | -61.66 | | <0.001 |
| *V*_c(max),m_ = 0.233+ 0.008*N*_m_ – 0.152*P*_m_ + 0.009*N*_m_ *P*_m_ | **0.54** | **-78.58** | **-65.31** | | <0.001 |
| *V*_c(max),m_ = min(– 0.287 + 0.037*N*_m_; 0.212 +0.309*P*_m_) | 0.52 | -74.25 | -59.98 | | <0.001 |
| Forest (Evergreen & Deciduous) | | | | | |
| *V*_c(max),m_ = – 0.069 + 0.025*N*_m_ | 0.48 | -117.16 | | -108.01 | <0.001 |
| *V*_c(max),m_ = 0.305 + 0.159*P*_m_ | 0.24 | -58.75 | | -49.60 | <0.001 |
| *V*_c(max),m_ = – 0.058 + 0.022*N*_m_ + 0.042*P*_m_ | 0.48 | -118.55 | | -106.35 | <0.001 |
| *V*_c(max),m_ = 0.179 + 0.013*N*_m_ – 0.164*P*_m_ + 0.007*N*_m_ *P*_m_ | **0.51** | **-125.24** | | **-109.99** | <0.001 |
| *V*_c(max),m_ = min(– 0.090 + 0.027*N*_m_; 0.349 – 0.194*P*_m_) | 0.49 | -120.90 | | -104.65 | <0.001 |
| Savanna (Deciduous) | | | | | |
| *V*_c(max),m_ = 0.102 + 0.018*N*_m_ | 0.30 | -84.97 | | -77.13 | <0.001 |
| *V*_c(max),m_ = 0.122 + 0.215*P*_m_ | 0.36 | -93.58 | | -85.73 | <0.001 |
| *V*_c(max),m_ = 0.032 + 0.010*N*_m_ + 0.152*P*_m_ | 0.42 | -102.07 | | -91.61 | <0.001 |
| *V*_c(max),m_ = 0.175 + 0.002*N*_m_ + 0.049*P*_m_ – 0.006*N*_m_ *P*_m_ | 0.42 | -101.40 | | -88.33 | <0.001 |
| *V*_c(max),m_ = min(– 0.026 + 0.029*N*_m_; – 0.006 + 0.347*P*_m_) | **0.48** | **-113.63** | | **-99.55** | <0.001 |
| Forest and Savanna (Deciduous and Evergreen) | | | | | |
| *V*_c(max),m_ = 0.038 + 0.021*N*_m_ | 0.42 | -231.72 | | -220.81 | <0.001 |
| *V*_c(max),m_ = 0.270 + 0.159*P*_m_ | 0.22 | -147.97 | | -137.06 | <0.001 |
| *V*_c(max),m_ = 0.011+ 0.017*N*_m_ + 0.074*P*_m_ | 0.46 | -249.48 | | **-234.94** | <0.001 |
| *V*_c(max),m_ = 0.113 + 0.013*N*_m_ – 0.006*P*_m_ + 0.003*N*_m_ *P*_m_ | **0.47** | **-250.68** | | -232.51 | <0.001 |
| *V*_c(max),m_ = min(– 0.061 + 0.028*N*_m_; 0.272 + 0.227*P*_m_) | 0.44 | -238.39 | | -219.22 | <0.001 |

**Table S3:** Comparisons of predictive models of mass based maximum electron transport rate (*V*_c(max),m_ 25 °C) based on leaf nitrogen and/or phosphorus content. Coefficients in black are significantly different from zero (*p* < 0.01); coefficients in red are not significantly different from zero (*p* > 0.05) and; coefficients in green are marginally different from zero (0.01 *> p* > 0.05).

| **Equation** | ***r*^2^** | ***AIC*** | | ***BIC*** | ***p*** |
| --- | --- | --- | --- | --- | --- |
| Forest Evergreen | | | | | |
| *J*_max,m_ = – 0.040 + 0.042*N*_m_ | 0.49 | 22.21 | | 28.01 | <0.001 |
| *J*_max,m_ = 0.577 + 0.256*P*_m_ | 0.35 | 34.38 | | 40.17 | <0.001 |
| *J*_max,m_ = 0.039 + 0.032*N*_m_ + 0.107*P*_m_ | 0.52 | 20.29 | | 28.02 | <0.001 |
| *J*_max,m_ = 0.753 + 0.007*N*_m_ – 0.604*P*_m_ + 0.023*N*_m_ *P*_m_ | **0.65** | **4.58** | | **14.24** | <0.001 |
| *J*_max,m_ = min(– 0.120 + 0.053N_m_; 0.436 + 0.447*P*_m_) | 0.59 | 13.36 | | 24.02 | <0.001 |
| Forest Deciduous | | | | | |
| *J*_max,m_ = – 0.344 + 0.054*N*_m_ | 0.50 | 68.48 | 76.44 | | <0.001 |
| *J*_max,m_ = 0.349 + 0.474*P*_m_ | 0.30 | 105.09 | 113.06 | | <0.001 |
| *J*_max,m_ = – 0.350 + 0.045*N*_m_ + 0.183*P*_m_ | 0.53 | 63.76 | 74.37 | | <0.001 |
| *J*_max,m_ = 0.541 + 0.013*N*_m_ – 0.532*P*_m_ + 0.024*N*_m_ *P*_m_ | **0.59** | **49.87** | **63.14** | | <0.001 |
| *J*_max,m_ = min(– 0.695 + 0.077*N*_m_; 0.298 + 0.616*P*_m_) | 0.53 | 64.07 | 78.34 | | <0.001 |
| Forest (Evergreen & Deciduous) | | | | | |
| *J*_max,m_ = – 0.229 + 0.050*N*_m_ | 0.50 | 88.38 | | 97.53 | <0.001 |
| *J*_max,m_ = 0.495 + 0.342*P*_m_ | 0.29 | 142.89 | | 152.04 | <0.001 |
| *J*_max,m_ = – 0.198 + 0.042*N*_m_ + 0.119*P*_m_ | 0.52 | 83.04 | | 95.24 | <0.001 |
| *J*_max,m_ = 0.624 + 0.013*N*_m_ – 0.596*P*_m_ + 0.024*N*_m_ *P*_m_ | **0.60** | **54.23** | | **69.48** | <0.001 |
| *J*_max,m_ = min(– 0.452 + 0.067*N*_m_; 0.341 + 0.556*P*_m_) | 0.53 | 78.24 | | 94.49 | <0.001 |
| Savanna (Deciduous) | | | | | |
| *J*_max,m_ = 0.108 + 0.036*N*_m_ | 0.40 | 8.65 | | 16.50 | <0.001 |
| *J*_max,m_ = 0.170 + 0.406*P*_m_ | 0.44 | 1.90 | | 9.75 | <0.001 |
| *J*_max,m_ = – 0.018 + 0.021*N*_m_ + 0.273*P*_m_ | 0.52 | -14.49 | | -4.03 | <0.001 |
| *J*_max,m_ = 0.365 – 0.001*N*_m_ – 0.002*P*_m_ + 0.015*N*_m_ *P*_m_ | 0.54 | -16.54 | | -3.46 | <0.001 |
| *J*_max,m_ = min(– 0.058 + 0.051*N*_m_; 0.023 + 0.587*P*_m_) | **0.58** | **-25.90** | | **-11.82** | <0.001 |
| Forest and Savanna (Deciduous and Evergreen) | | | | | |
| *J*_max,m_ = – 0.044 + 0.042*N*_m_ | 0.49 | 97.97 | | 108.88 | <0.001 |
| *J*_max,m_ = 0.434 + 0.328*P*_m_ | 0.26 | 203.36 | | 214.27 | <0.001 |
| *J*_max,m_ = – 0.100 + 0.036*N*_m_ + 0.153*P*_m_ | 0.54 | 73.73 | | 88.27 | <0.001 |
| *J*_max,m_ = 0.368 + 0.017*N*_m_ – 0.212*P*+ 0.014*N*_m_ *P*_m_ | **0.57** | **53.87** | | **72.04** | <0.001 |
| *J*_max,m_ = min(– 0.203 + 0.055*N*_m_; 0.458 + 0.450*P*_m_) | 0.51 | 89.40 | | 108.58 | <0.001 |

**Table S4.** Comparisons of predictive models of mass based maximum electron transport rate (*J*_max,m_ 25 °C) based on leaf nitrogen and/or phosphorus content. Coefficients in black are significantly different from zero (*p* < 0.01); coefficients in red are not significantly different from zero (*p* > 0.05) and; coefficients in green are marginally different from zero (0.01 > *p* > 0.05).

**Fig.** **S5** *V*_max_ on a mass basis. Plots of residuals versus observed values of leaf mass per unit area (*M*_a_); area based nutrients and foliar N:P ratios. Members of the Fabaceae family: closed squares; all other species: open circles.

**Fig. S6** *J*_max_ on a mass basis: Plots of residuals versus observed values of leaf mass per unit area (*M*_a_); area based nutrients and foliar N:P ratios. Members of the Fabaceae family: closed squares; all other species: open circles.
